# The host metabolite D-serine contributes to bacterial niche specificity through gene selection

**DOI:** 10.1038/ismej.2014.242

**Published:** 2014-12-19

**Authors:** James PR Connolly, Robert J Goldstone, Karl Burgess, Richard J Cogdell, Scott A Beatson, Waldemar Vollmer, David GE Smith, Andrew J Roe

**Affiliations:** 1 Institute of Infection, Immunity and Inflammation, University of Glasgow, Glasgow, UK; 2 School of Life Sciences, College of Medical, Veterinary and Life Sciences, University of Glasgow, Glasgow, UK; 3 Institute of Molecular Cell and Systems Biology, University of Glasgow, Glasgow, UK; 4 School of Chemistry and Molecular Biosciences and Australian Infectious Diseases Research Centre, University of Queensland, St Lucia, Queensland, Australia; 5 Centre for Bacterial Cell Biology, Institute for Cell and Molecular Biosciences, Newcastle University, Newcastle upon Tyne, UK; 6 Moredun Research Institute, Pentlands Science Park, Bush Loan, Edinburgh, Midlothian, UK

## Abstract

*Escherichia coli* comprise a diverse array of both commensals and niche-specific pathotypes. The ability to cause disease results from both carriage of specific virulence factors and regulatory control of these *via* environmental stimuli. Moreover, host metabolites further refine the response of bacteria to their environment and can dramatically affect the outcome of the host–pathogen interaction. Here, we demonstrate that the host metabolite, D-serine, selectively affects gene expression in *E. coli* O157:H7. Transcriptomic profiling showed exposure to D-serine results in activation of the SOS response and suppresses expression of the Type 3 Secretion System (T3SS) used to attach to host cells. We also show that concurrent carriage of both the D-serine tolerance locus (*dsdCXA*) and the locus of enterocyte effacement pathogenicity island encoding a T3SS is extremely rare, a genotype that we attribute to an ‘evolutionary incompatibility' between the two loci. This study demonstrates the importance of co-operation between both core and pathogenic genetic elements in defining niche specificity.

## Introduction

*Escherichia coli* is a diverse Gram-negative bacterium that comprises several subgroups (A, B1, B2, C, D, E and F) based on genetic phylogeny (Wirth *et al.*, 2006; Sims and Kim, 2011). The genetic variation between these groups is vast with the ‘core' genome consisting of a mere ∼2000 genes in contrast to the *E. coli* pan genome of ∼18 000 genes (Van, Elsas *et al.*, 2010). Within this variation, the acquisition and loss of genomic islands between strains, both in the context of virulence and environmental adaptation, plays a key role in defining niche specificity (Dobrindt *et al.*, 2004). Furthermore, the function of the core genome in defining a strain's metabolic capacity contributes to the ability of *E. coli* strains to occupy distinct ecological niches (Touchon *et al.*, 2009; Van, Elsas *et al.*, 2010; De Muinck *et al.*, 2013). Despite the vast array of research that has been carried out to understand the physiology, biochemistry and genetics of both pathogenic and commensal *E. coli* strains, identifying the specific attributes that define a strain's favored niche remains challenging.

*E. coli* within phylogroup B2 provide intriguing examples of niche diversification from intestinal commensalism and pathogenesis to strains highly virulent in the urinary tract (uropathogenic *E. coli* or UPEC) and other extra-intestinal sites (Kaper *et al.*, 2004; Brzuszkiewicz *et al.*, 2006; Wiles *et al.*, 2009). The latter are termed extraintestinal pathogenic *E. coli* because of their ability to cause disease beyond the gastrointestinal tract. Through the acquisition of combinations of virulence factors, such as fimbrial adhesins, capsule and iron acquisition systems, UPEC strains compete and thrive in the bladder, an organ very different to the intestine. Despite these defining features, UPEC strains continue to passage through the digestive system very successfully, without any apparent loss in intestinal fitness (Chen *et al.*, 2013). In contrast, intestinal pathogenic *E. coli* such as enterohaemorrhagic *E. coli* (EHEC) O157:H7, are highly niche-specific and are very rarely associated with the colonization of distal sites (Kaper *et al.*, 2004; Wong *et al.*, 2011). This is somewhat surprising when one considers that, at least *in vitro*, EHEC have a capacity to bind and attach to a wide variety of receptors present on a range of cell types including red blood cells, lung, cervical, as well as intestinal (Shaw *et al.*, 2002; Torres and Kaper, 2003; Holmes *et al.*, 2012). It is therefore imperative to understand why EHEC are limited to the gastrointestinal tract and what factors might be acquired to allow dissemination to new niches.

A key trait of a successful pathogen is the ability to compete with the established microbial residents to facilitate an infection (De Muinck *et al.*, 2013). Recent work has highlighted the importance of metabolism in this process. Different *E. coli* pathotypes often utilize discrete sets of sugars ensuring they occupy distinct sites within the gastro-intestinal tract (Meador *et al.*, 2014). In addition to sugar metabolism, one interesting difference between UPEC and EHEC strains is carriage of the locus for D-serine metabolism, *dsdCXA* (Cosloy and McFall, 1973; Nørregaard-madsen and Fall, 1995). This locus codes for a D-serine deaminase (DsdA), a D-serine inner membrane transporter (DsdX) and an essential LysR-type regulator of the system (DsdC). The role of *dsdCXA* has been defined to detoxify UPEC strains from inhibitory concentrations of the host metabolite D-serine encountered primarily in the urinary tract during infection (reported concentration range of ∼28 to ∼1 mM in urine) (Nørregaard-madsen and Fall, 1995; Anfora *et al.*, 2007). Indeed, UPEC strains can metabolize D-serine as a sole carbon source (Anfora and Welch, 2006). Conversely, studies have shown that 95% of diarrheagenic *E. coli* strains tested fail to grow on D-serine as a sole carbon source. Prototypic *E. coli* O157:H7 isolates such as EDL933 contain a truncated *dsdCXA* locus, resulting in *dsdC* and a portion of *dsdX* being substituted with the *csrRAKB* sucrose utilization locus occurring at a chromosomal recombination hotspot (Jahreis *et al.*, 2002; Moritz and Welch, 2006). The acquisition of the ability to utilize sucrose provides an obvious and tangible advantage to the pathogen, but the loss of *dsdCX* is obscure. There is a clear potential for EHEC strains to encounter D-serine in the environment, for example, through dietary intake (Friedman, 1999; Csapo, 2009) or even contact with urine post-fecal shedding from the host. Also, recent studies have demonstrated the ability of certain bacteria to naturally produce non-canonical D-amino acids (D-AAs) (Lam *et al.*, 2009; Cava, de Pedro, *et al.*, 2011).

The regulatory roles of D-AAs in bacteria have also been explored with implicated functions in spore germination in *Bacillus* species and recently, key roles in cell wall reorganization in both *Vibrio cholerae* and *Bacillus subtilis* (Lam *et al.*, 2009; Cava, de Pedro, *et al.*, 2011; Cava, Lam, *et al.*, 2011). D-serine has been implicated to harbor a regulatory role in UPEC virulence during urinary tract infection (Roesch *et al.*, 2003; Anfora *et al.*, 2007).

On the basis of the aforementioned data, involving the role of D-AAs in signaling and showing the truncation of the *dsdCXA* locus in EHEC, we hypothesized that D-serine could influence niche selectivity of different *E. coli* pathotypes. We found that D-serine alone caused repression of the Type 3 Secretion System (T3SS) and induced the SOS response (Kenyon and Walker, 1980; Michel, 2005). These phenotypes were entirely independent of one another, highlighting the vast array of regulatory roles D-AAs can play in bacteria. We also show that carriage of both *dsdCXA* and the locus of enterocyte effacement (LEE) genes encoding a T3SS is extremely rare, a genotype that we attribute to an ‘evolutionary incompatibility' between the two loci. This study demonstrates the importance of evolutionary co-operation between both core and pathogenic genetic elements in defining niche specificity. Overall, we show that D-serine influences both gene content and regulation of critical virulence factors in pathogenic *E. coli.*

## Materials and methods

### Bacterial strains, plasmids and growth conditions

All wild-type and mutant bacterial strains used in this study are listed in Supplementary Table S2 and plasmids used are listed in Supplementary Table S3. Mutant strains TUV93-0 Δ*yhiF*, CFT073 Δ*dsdA* and CFT073 Δ*dsdX*Δ*cycA* as well as complementation constructs (p*yhiF*, p*dsdA* and p*dsdX*) were generated as described elsewhere and generously supplied as gifts for use in this study (Anfora and Welch, 2006; Tree *et al.*, 2011). Single colonies of bacteria were inoculated into 5 ml LB broth containing the appropriate antibiotics where specified (ampicillin 100 μg ml^−1^; kanamycin 50 μg ml^−1^; chloramphenicol 25 μg ml^−1^) and cultured overnight at 37 °C, 200 r.p.m. Overnight cultures were used to inoculate pre-warmed MEM-HEPES (Sigma-Aldrich, St Louis, MO, USA; cat # m7278) at an OD^600^ of 0.05 and samples were cultured subsequently at 37 °C, 200 r.p.m. D-AAs for screening were purchased from Sigma-Aldrich and supplemented into MEM-HEPES at a concentration of 1 mM unless otherwise stated. Mitomycin C was used as a positive control for inducing the SOS response at a concentration of 5 μM.

### *In vitro* GFP reporter-fusion assay of LEE promoter activity

Transcriptional GFP reporter-fusions for the LEE1–3 and *tir* promoters (PLEE1:GFP, PLEE2:GFP, PLEE3:GFP and P*tir*:GFP) were used to measure the effects of different D-AAs expression on the T3SS. A P*rpsM*::GFP reporter was used to control for any variation in expression of housekeeping genes. Reporter plasmids used in this study were generated as previously described (Roe *et al.*, 2003) and are listed in Supplementary Table S3. Aliquots of bacterial culture were taken at regular intervals and transferred to a black 96-well plate for GFP fluorescence measurement on a FLUOstar Optima Fluorescence Plate Reader (BMG Labtech, Jena, Germany). GraphPad Prism software version 5.0c (GraphPad Software, San Diego, CA, USA) was used to generate a standard curve of OD^600^ versus fluorescence (relative fluorescence unit) and obtain values at OD^600^ 0.7 for comparison between samples.

### SDS-PAGE and immunoblot analysis of type III secreted proteins

Analysis of T3SS proteins was carried out as described previously (Tree *et al.*, 2011). Cultures of bacteria in MEM-HEPES (as above) were grown to an OD^600^ of 0.7 and supernatants were obtained by centrifugation at 4000 r.p.m. for 15 min. Cell pellets were retained for analysis of whole cell proteins and were lysed using BugBuster Protein Extraction buffer (Merck, New Jersey, USA). Supernatants were syringe-filtered (0.45 μm) and secreted proteins (Sec) were precipitated overnight using 10% v/v TCA (Sigma-Aldrich) at 4 °C. Secreted proteins were harvested by centrifugation at 4000 r.p.m. (4 °C) for 1 h. Sec pellets were resuspended in Tris-HCl (pH 8.0) and 5 μl aliquots were analysed by SDS-PAGE using the Novex system (Invitrogen, Carlsbad, CA, USA). Primary antibodies used for immunoblotting were for EspD (1/6000), Tir (1/2000), RecA (1/4000) and GroEL (1/20000).

### Total RNA extraction and mRNA enrichment

Bacterial cultures were grown as above and mixed with two volumes of RNAprotect reagent (Qiagen, Valencia, CA, USA), incubating for 5 min at room temperature. Cell pellets were harvested by centrifugation and total RNA was extracted using an RNeasy kit (Qiagen). Genomic DNA was removed post extraction using a TURBO DNase kit (Ambion, Carlsbad, CA, USA) and samples were enriched for mRNA using a MICROBexpress mRNA enrichment kit (Ambion). Samples for RNA-seq analysis were QC tested for integrity and rRNA depletion using an Agilent Bioanalyzer 2100 (University of Glasgow, Polyomics Facility).

### RNA-seq transcriptome generation and data analysis

cDNA synthesis and sequencing was performed at the University of Glasgow Polyomics Facility on an Illumina Genome Analyser IIx (Illumina, San Diego, CA, USA), using 70 bp single-end reads and six samples per lane. Raw reads were QC checked using FastQC (Babraham Bioinformatics, Cambridge, UK) and trimmed accordingly using CLC Genomics Workbench (CLC Bio, Aarhus, Denmark). Data were normalized and analysed for differentially expressed genes using the Bioconductor packages DEseq and EdgeR (Anders and Huber, 2010; Robinson *et al.*, 2010) with raw reads mapped to the EDL933 reference genome (NCBI accession number: NC_002655.2). The sequence reads reported in this paper have been deposited in the European Nucleotide Archive under study PRJEB7974 (samples ERS627411 and ERS627412). Differentially expressed genes were identified using a significance cutoff of ⩽0.05 and changes of interest were validated using qRT-PCR. An experiment investigating the global effects of D-serine on *E. coli* O157:H7 consisted of three biological replicates of WT TUV93-0 cultured in MEM-HEPES and two biological replicates of TUV93-0 cultured in MEM-HEPES supplemented with 1 mM D-serine.

### Quantitative real time-PCR (qRT-PCR)

Validation of differentially expressed genes identified *via* RNA-seq was carried out by qRT-PCR using KAPA SYBR FAST Universal qRT-PCR master mix (KAPA Biosystems, Woburn, MA, USA) and M-MLV Reverse Transcriptase (Promega, Madison, WI, USA). Total RNA was extracted as above and was quantified using a Nanodrop 2000 (Thermo Scientific, Waltham, MA, USA). Samples for comparison were normalized to a concentration of 50 ng μl^−1^ using RNAse free TE buffer (Ambion). qRT-PCR analysis was performed using a two-step reaction; cDNA synthesis (37 °C for 15 min and 95 °C for 10 min) followed by qRT-PCR (40 cycles of 95 °C for 10 s, 55 °C for 30 s). Individual reactions were performed in triplicate to eliminate technical variance and reaction for each gene to be analysed was performed in biological triplicate. qRT-PCR reactions were carried out using the ECO Real-Time PCR System (Illumina) according to the manufacturers specifications and the data were analysed according to the 2^−ΔΔCt^ method (Livak and Schmittgen, 2001) using the housekeeping gene *gapA* as an internal control. All primers used are listed in Supplementary Table S4.

### HeLa cell adhesion assay and microscopy

Coverslips were seeded with 4 × 10^4^ HeLa cells in a multi-well plate and incubated overnight in MEM-HEPES at 37 °C with 5% CO2. TUV93-0 transformed with a pRFP plasmid were used for adhesion assays. Bacterial cultures for infection were grown in MEM-HEPES at 37 °C until at an OD^600^ of 0.6. Seeded cells were washed with fresh media and infected with 100 μl bacterial culture (adjusted to OD^600^ of 0.1) in 500 μl fresh MEM-HEPES with or without 1 mM D-serine. Plates were centrifuged at 400 r.p.m. for 3 min and incubated at 37 °C with 5% CO2 for 2 h. Wells were washed with fresh media to remove unbound bacteria and incubated as above for a further 3 h. Wells were washed three times with sterile PBS before fixing for 20 min with 250 μl PFA (2%). Wells were washed again with PBS. Wells were incubated for 5 min with 250 μl of Triton X-100 (0.5%) and washed. Host cell actin was stained with Phalloidin-488 (Invitrogen) for 1 h. Wells were washed a final time before mounting on microscope slides with fluorescent mounting medium (Dako, Cambridge, UK). Slides were imaged using a Zeiss M1 Axioimager microscope and data were acquired and deconvoluted using the Zen Pro software (Zeiss, Jena, Germany). Adhesion assays were performed in triplicate.

### Growth on D-serine as a sole carbon source

Assessment of a strain's ability to grow using D-serine as a sole carbon source was carried out using MOPS minimal media agar plates supplemented with D-serine, as described previously (Anfora and Welch, 2006). MOPS minimal media consisted of 1.32 mM K_2_HPO_4_, 9.52 mM NH_4_Cl, 0.523 mM MgCl_2_, 0.276 mM K_2_SO_4_, 10 μM FeSO_4_, 0.5 μM CaCl_2_, 50 mM NaCl, 40 mM MOPS, 4 mM Tricine, 3 nM (NH_4_)_6_(MO_7_)_24_, 0.4 μM H_2_BO_3_, 30 nM CoCl_2_, 10 nM CuSO_4_, 80 nM MnCl_2_ and 10 nM ZnSO_4_. Plates were supplemented with either 43.4 mM glycerol (as a positive control) or 4.76 mM D-serine (as a sole carbon source) as previously described elsewhere (Anfora and Welch, 2006). Strains of interest were streaked and grown at 37 °C on MOPS plates.

### Extraction of whole-cell metabolites and HPLC analysis

Preparation of metabolites was adapted from Creek *et al.* (2011). Bacterial cultures were grown to an OD^600^ of 0.6 in MEM-HEPES, supplemented with and without 1 mM D-serine, and were subsequently diluted to an OD^600^ of 0.1 in fresh, warm MEM-HEPES to a final volume of 40 ml. Cultures were quenched by rapid submersion in ethanol and dry ice to cool samples to 4 °C. Cells were then centrifuged at 3500 r.p.m. (4 °C) for 10 min. Bacterial cell pellets were resuspended in 1 ml of supernatant and transferred to an ice-cold 1.5 ml microcentrifuge tube before obtaining the final cell pellet at 3500 r.p.m. (4 °C) for 5 min. The supernatant was completely removed at this stage and metabolites were extracted from the cell pellet by addition of 200 μl of chloroform/methanol/water (1:3:1) at 4 °C and tubes were vortexed vigorously for 1 h at 4 °C. Cellular debris and precipitate was removed by a final centrifugation at 13 000 r.p.m. (4 °C) for 5 min. Supernatant was transferred to a fresh tube and stored at −80 °C until LC-MS analysis.

Samples were separated on a Chirobiotic T2 column (2 mm × 25 cm; Sigma-Aldrich) using an isocratic flow at 85% ethanol/15% water at 200 μl min^−1^. Amino acids were detected using atmospheric pressure chemical ionization in an Orbitrap Elite (Thermo Scientific) at 70 000 resolution. Source settings were configured as described previously (Desai and Armstrong, 2004). Analysis was performed using the ExactFinder software (Thermo Scientific).

### Bioinformatic analysis of *dsdCXA* and the LEE carriage in *E. coli*

The nucleotide sequences for 159 core genes in *E. coli* were elaborated as described in a recent publication (R. Goldstone, in preparation). Briefly, at each iteration, the core gene set, initialized as the nucleotide sequence for genes present in MG1655, was aligned to the next *E. coli* genome sequence using blastn (Altschul *et al.*, 1990). Genes aligning at >70% identity and >80% of the length of the coding sequence were retained in the core gene set for use in the next iteration. This analysis resulted in the identification of 159 genes. The nucleotide sequences of each core gene were extracted from the *E. coli* genomes, aligned by Muscle (Edgar, 2004), concatenated, and a maximum likelihood tree calculated using PhyML (Guindon and Gascuel, 2003) under the general time reversible model of nucleotide substitution. Dendrograms were visualised using the APE package (Paradis *et al.*, 2004) implemented in R (R Development Core Team, 2012).

The amino acid sequences for genes present within O-island 148, encoding the LEE T3SS system were collected from the sequence for EDL933 (NCBI accession number: NC_002655.2), and amino acid sequences for DsdX, DsdC and DsdA were collected from the sequence for CFT073 (NCBI accession number: NC_004431.1). These sequences were iteratively aligned against the *E. coli* genomes using tblastn and called as present in a genome if the protein sequence aligned at greater than 70% identity over greater than 80% to the translated nucleotide sequence of the genome in question. The distribution of the genes across the *E. coli* core-genome dendrogram was visualized using the Diversitree package (Fitzjohn, 2012) implemented in R (R Development Core Team, 2012).

For statistical analysis to take account of genes incorrectly labelled as ‘absent' because of the splitting of coding sequences across contigs, the LEE was called as ‘present' if an isolate possessed 21 or more of the following: *espF, Z5102, escF, Z5014, espB, espD, espA, sepL, escD, eae, Z5111, tir, Z5113, Z5114, Z5115, sepQ, Z5117, Z5118, escN, escN, Z5121, sepZ, Z5123, escJ, Z5125, escC, cesD, Z5128, Z5129, Z5131, escU, escT, escS, escR, Z5136, Z5137, Z5138, Z5139, Z5140, Z5142* and *Z5143*. *dsdCXA* was called as present only if all three of *dsdX, dsdC and dsdA* were identified. Statistical significance for the association between *dsdCXA* and the LEE was evaluated by Fisher's exact test.

## Results

### Global analysis of the effects of D-serine on EHEC

**Figure 1:**
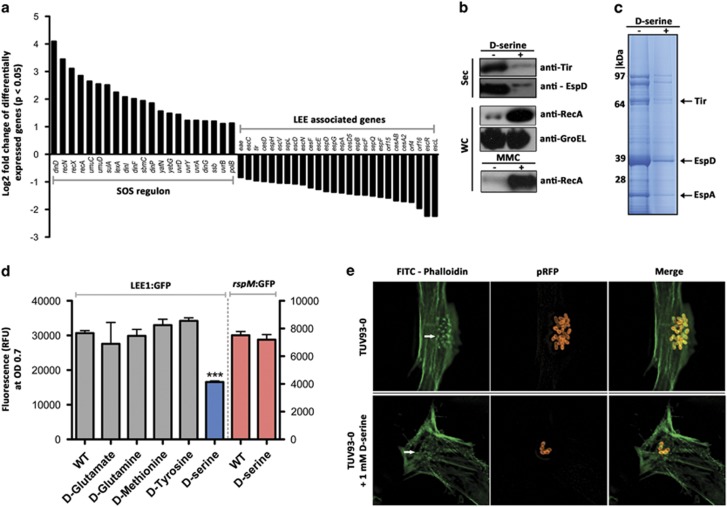
D-serine induces the SOS response and represses the T3SS in *E. coli* O157:H7. (**a**) Co-ordinated induction of the SOS response and repression of the LEE in TUV93-0 cultured in MEM-HEPES supplemented with D-serine as identified by RNA-seq analysis. Histogram bars indicate the Log^2^ fold change of differentially expressed genes wit a *P*-value of ⩽0.05. (**b**) Immunoblot analysis of secreted proteins (Sec) and whole-cell lysate (WC) from TUV93-0 cultured in MEM-HEPES alone (−) and supplemented with 1 mM D-serine (+). Tir, EspD and RecA were used as markers for type 3 secretion and the SOS response. Equal amount of Sec fractions were analysed in − and + lanes. GroEL was used as a loading control for the WC fractions and these corresponded to the Sec fractions. Cultures supplemented with 5 μM mitomycin C to induce the SOS response were used as a positive control. (**c**) SDS-PAGE analysis of the type 3 secretion profile from TUV93-0 cultured in MEM-HEPES alone (−) and supplemented with 1 mM D-serine (+). Protein bands corresponding to Tir, EspD and EspA were identified by MS-MS analysis and are indicated with black arrows. (**d**) Screening the effects of multiple D-AAs on LEE1 expression using a GFP tagged LEE1 promoter fusion reporter (PLEE1:GFP). A subset of all D-AAs tested (D-glutamate, D-glutamine, D-methionine, D-tyrosine and D-serine) is shown with D-serine alone showing a significant decrease in LEE1 expression (blue bar). *** denotes *P*⩽0.001 calculated from three biological replicates. An *rpsM* GFP reporter was used as a housekeeping control (P*rpsM*:GFP) and is indicated by red bars on the right *y* axis. Relative fluorescence units (RFU) were derived from a standard curve of optical density at 600 nm (OD^600^) measured over time. Bacteria were cultured in MEM-HEPES to promote expression of the T3SS. (**e**) Wide-field fluorescence microcopy images of HeLa cells infected with TUV93-0 (with and without the addition of 1 mM D-serine to the growth medium). Host HeLa cells were stained with FITC-Phalloidin (green) and bacteria with carrying a constitutively expressed RFP construct (red). Actin condensation, corresponding to pedestal formation, is indicated by a white arrow.

Previous work has demonstrated D-AAs can play an important role in the regulation of cellular processes and that D-serine is particularly important for UPEC pathogenesis (Anfora *et al.*, 2007; Cava, Lam, *et al.*, 2011). Our hypothesis was that D-serine might also play an important role in regulating gene expression in *E. coli* O157:H7. To investigate this, we used RNA-sequence analysis (RNA-seq), which provided us with a global view into the effects of D-serine on gene expression (Wang *et al.*, 2009). A concentration of 1 mM D-serine was used as this represents a physiologically relevant level as previously reported (Anfora *et al.*, 2007). We also found concentrations upwards of 2 mM D-serine had no significant effects on growth or viability in the media tested (data not shown). Significantly (*P*⩽0.05) up- and downregulated genes were identified using the DESeq and EdgeR packages of Bioconductor (Anders and Huber, 2010; Robinson *et al.*, 2010). The most strongly upregulated genes included members of the SOS-regulon (Fernández De Henestrosa *et al.*, 2000; Salgado *et al.*, 2013). This comprised 21 genes including the well-characterized SOS components *lexA*, *recA* and *sulA* (Figure 1a; Supplementary Table S1). These genes are normally associated with a global response to DNA-damage (Kenyon and Walker, 1980; Michel, 2005) and the addition of a single D-AA seemed inconsistent with such detrimental effects. These results were confirmed by immunoblotting for the SOS anti-repressor RecA, which was markedly upregulated compared with the untreated control (Figure 1b) and RNA-seq data were also validated by qRT-PCR (Supplementary Figure 1).

Addition of D-serine significantly reduced the expression of 26 out of the 42 genes that comprise the LEE, a horizontally acquired pathogenicity island which encodes a major virulence factor, the T3SS, across five operons, LEE1 to LEE5 (Supplementary Table S1) (McDaniel *et al.*, 1995; Croxen and Finlay, 2010; Büttner, 2012). The LEE-encoded genes downregulated included those encoding for membrane structural components, the needle proteins and secreted effectors (Figure 1a). The normal function of the LEE is to translocate bacterial effector proteins into host cells and subvert normal cell function (Büttner, 2012). Secretion of several of these effector proteins can be readily assayed by growing EHEC in media that induces secretion in the absence of host cells (Tree *et al.*, 2011). Separation by SDS-PAGE and visualization with Coomassie blue allows several well-characterized proteins to be detected including Tir, EspA and EspD. Addition of 1 mM D-serine caused a marked reduction in the production of effector proteins (Figure 1c), a phenotype that was consistent with downregulation of the LEE. The results were confirmed by immunoblotting for both Tir and EspD, which showed a greater than sevenfold reduction in effector protein secretion after addition of D-serine (Figure 1b). As a control, the corresponding whole-cell fractions were probed for GroEL, which showed no differences (Figure 1b).

### Downregulation of the T3SS is D-serine-specific and affects host cell binding

To test whether the phenotype was specific to D-serine, we analysed the effects of numerous D-AAs on expression of the LEE. Addition of 1 mM of D-glutamate, D-glutamine, D-methionine, D-tyrosine, D-valine, D-tryptophan, D-phenylalanine and D-leucine had no effect on LEE expression, as determined by measuring transcription of the LEE master regulator Ler (Figure 1d) (Elliott *et al.*, 2000). In contrast, addition of 1 mM D serine reduced *ler* expression by 54% (*P*⩽0.0001). As a control, we tested transcription from the promoter for the ribosomal protein, RpsM, which was unaffected (Figure 1d). Moreover, L-serine had no effect, highlighting the specificity of the phenotype solely to D-serine. Finally, we found D-serine to be significantly detrimental to LEE expression at concentrations of ⩾100 μM in MEM-HEPES (Supplementary Figure 2).

Given that D-serine selectively downregulated the T3SS, we then tested the ability of EHEC to bind and intimately attach to host cells. WT bacteria use their T3SS to translocate effector proteins into the host cell resulting in distinctive areas of condensed host-cell actin, called pedestals (Figure 1e) (Croxen and Finlay, 2010). Addition of 1 mM D-serine during infection does not affect bacterial growth rate in the media used but resulted in 77% fewer infected host cells relative to the untreated control (*P*<0.001). Moreover, D-serine also reduced the bacterial numbers on successfully infected host cells by 62% (*P*<0.01) and fewer of the attached bacteria formed pedestals (Figure 1e and Supplementary Figure 3A). As a control, an isogenic TUV93-0 Δ*tir* strain was used. As expected, this failed to translocate Tir and form pedestals (Supplementary Figure 3B). The reduction in LEE expression at D-serine concentrations of ⩾100 μM implied that the levels in the gastro-intestinal tract would be significantly lower to permit LEE functionality. Measurement of D-serine in the colon of five BALB/C mice revealed levels of ∼1 μM relative to a D-serine standard (data not shown) some 100 times lower than the concentration required for inhibition of LEE expression.

### T3SS repression by D-serine is independent of the SOS response

**Figure 2:**
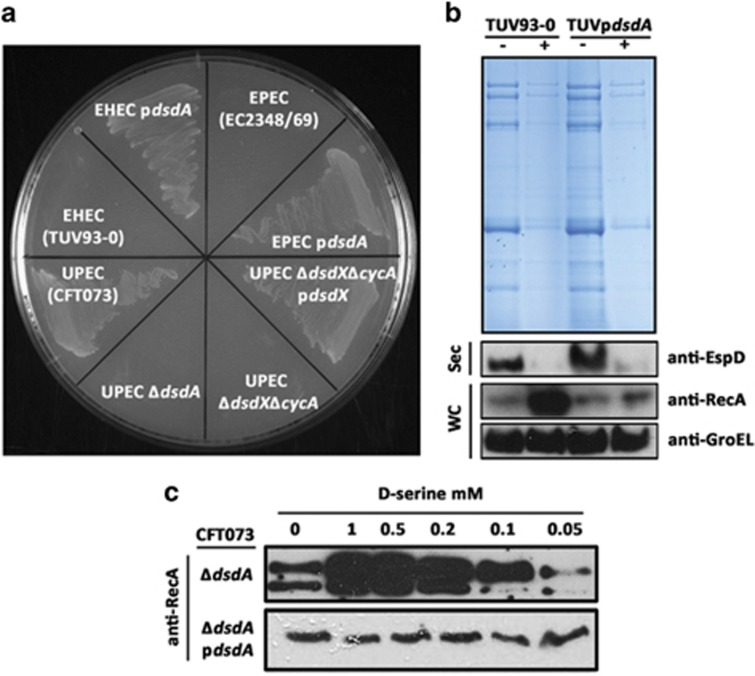
The effects of D-serine accumulation on growth, the SOS response and the T3SS. (**a**) Growth of various *E. coli* strains on MOPS minimal agar plates containing D-serine as a sole carbon source. WT UPEC (CFT073) or EHEC (TUV93-0) and EPEC (EC234/69) complemented with p*dsdA* were capable of growing on D-serine as a sole carbon source. WT EHEC and EPEC as well as a UPEC Δ*dsdA* mutant were unable to grow. A UPEC Δ*dsdX/cycA* D-serine transporter double mutant was also unable to grow. (**b**) SDS-PAGE and immunoblot analysis of secreted proteins from TUV93-0 and TUV93-0 complemented with *dsdA* from UPEC CFT073 in trans (TUV*pdsdA*). Strains were cultured in MEM-HEPES alone (−) and supplemented with 1 mM D-serine (+). Immunoblot analysis of EspD levels from the secreted fraction (Sec) and RecA and GroEL from the whole-cell lysate (WC) are displayed. Equal amounts of Sec fraction were loaded for each lane and WC fractions from the corresponding Sec fractions were used to indicate identical total protein levels between samples. GroEL was used as a loading control. (**c**) Immunoblot analysis of RecA expression in UPEC Δ*dsdA* over five increasing D-serine concentrations. Complementation with p*dsdA* resulted in no increased RecA expression.

Previous work has demonstrated that the majority of EHEC cannot grow on D-serine as a sole carbon source, the assumption being that these strains do not express a deaminase capable of metabolizing this amino acid (Moritz and Welch, 2006). Correspondingly, the EHEC strain used in this study (TUV93-0) failed to grow on MOPS minimal agar plates containing D-serine as a sole carbon source (Figure 2a). We postulated that the accumulation of intracellular D-serine resulted in expression of the SOS response. This hypothesis was tested in two ways. Firstly, a plasmid-borne copy of D-serine deaminase (p*dsdA*) was introduced into EHEC and secondly, the UPEC strain CFT073 was deleted for *dsdA*. In both cases, the SOS response and the level of intracellular D-serine were measured. Transformation of EHEC with p*dsdA*, enabled growth on D-serine plates (Figure 2a) showing clearly that the strain was now capable of actively metabolizing D-serine. Intracellular D-serine levels of EHEC p*dsdA* were reduced>sevenfold compared with the WT, consistent with D-serine breakdown (Supplementary Figure 4A and B). Interestingly, the expression of RecA levels in EHEC p*dsdA* showed that they were restored to the same levels as seen in the absence of D-serine (Figure 2b). Furthermore, intracellular accumulation of D-serine was evident in a UPEC Δ*dsdA* mutant and this resulted in strong RecA expression, indicating activation of the SOS response (Figure 2c). In contrast, analysis of the secreted protein profile and immunoblotting for EspD showed that even when EHEC can metabolize D-serine, the T3SS remains inhibited and production of effector proteins (Figure 2b) and the ability to attach to host cells (Supplementary Figure 3C) is reduced. These data show that the SOS response is dependent on intracellular accumulation of D-serine and that repression of the T3SS by D-serine occurs even in the absence of the SOS response.

### D-serine regulates the T3SS through yhiF and ihfA

**Figure 3:**
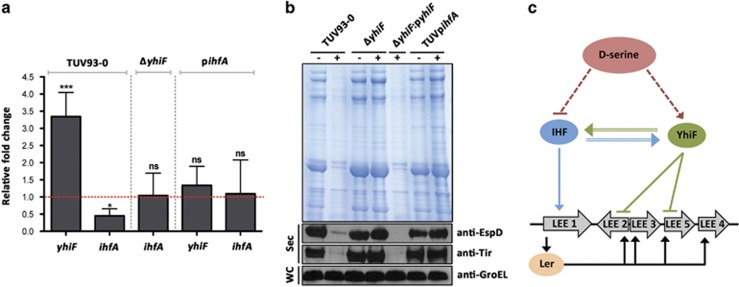
The mechanism of D-serine repression of the T3SS in *E. coli* O157:H7. (**a**) qRT-PCR validation of differentially expressed LEE regulators (*yhiF* and *ihfA*) in response to D-serine identified by RNA-seq. The expression of *yhiF* and *ihfA* in response to D-serine was investigated by analysing relative mRNA transcript levels of each regulator in the corresponding complementation or knockout background. The wild-type TUV93-0, Δ*yhiF* and p*ihfA* backgrounds are indicated above the bars and are separated by gray dashed lines. The red dashed line indicates relative baseline expression, with genes expressed above this being upregulated and genes expressed below this being downregulated. ns, * and *** denote no significance, *P*⩽0.05 and *P*⩽0.001, respectively, calculated from three biological replicates. (**b**) SDS-PAGE and immunoblot analysis of secreted proteins (Sec) from TUV93-0, Δ*yhiF*, Δ*yhiF* complemented with *yhiF* in trans (Δ*yhiF*:p*yhiF*) and TUV93-0 complemented with *ihfA* in trans (TUV:p*ihfA*). Strains were cultured in MEM-HEPES alone (−) and supplemented with 1 mM D-serine (+). Equal amounts of the Sec fraction were loaded for each lane and whole-cell lysate (WC) fractions from the corresponding Sec fractions were used to indicate identical total protein levels between samples. GroEL was used as the loading control. (**c**) Regulatory model of D-serine repression on the LEE pathogenicity island *via* YhiF and IhfA. Arrows indicate positive regulation, whereas blunt end lines indicate negative regulation. Solid lines indicate a direct signal, double lines indicate cross regulation and dashed lines indicate an unknown signal. Expression of LEE1 to LEE5 operons is driven through Ler. IHF directly upregulates the LEE1 promoter, whereas YhiF represses the LEE2 and LEE5 promoters. Wild-type TUV93-0 supplemented with D-serine leads to downregulation of *ihfA* and upregulation of *yhiF. ihfA* and *yhiF* respond co-operatively in response to D-serine and differential expression of both regulators is required for D-serine-mediated repression of the LEE.

Examination of the RNA-seq data revealed differential expression of known regulators that might explain the repression of the LEE. We identified that D-serine differentially affected the expression of two DNA-binding transcriptional activators, IhfA (Integration Host Factor alpha-subunit) and the GAD acid stress response regulator YhiF. Previous work has shown Integration Host Factor (IHF) directly binds the LEE1 promoter as a positive regulator of the T3SS. YhiF is a member of the LuxR family of transcriptional regulators and has been shown to negatively regulate the LEE2 and LEE5 promoters (Friedberg *et al.*, 1999; Tatsuno *et al.*, 2003; Tree *et al.*, 2011). The upregulation of *yhiF* and downregulation of *ihfA* were verified using qRT-PCR analyses (Figure 3a). As both IhfA and YhiF have been linked to regulation of the LEE, we evaluated their contribution to the D-serine phenotype. Given that *yhiF* was upregulated in the presence of D-serine, we hypothesized that a Δ*yhiF* deletion strain might be less sensitive to this amino acid. Similarly, as *ihfA* was downregulated in response to D-serine, the prediction is that constitutive complementation of this activator might similarly reduce the repressive effects. As demonstrated in Figure 3b, deletion of *yhiF* provided protection from the repressive effects of D-serine. This phenotype could be complemented by transformation of the mutant with a plasmid-borne copy of *yhiF*. Similarly, EHEC transformed with a plasmid constitutively expressing *ihfA* (p*ihfA*) were shown to be insensitive to D-serine addition. Furthermore, to investigate whether YhiF and IhfA were cross-regulated in response to D-serine, qRT-PCR analysis of each transcript in the corresponding deletion or complementation background was carried out (Figure 3a). These results revealed that differential expression of these regulators in response to D-serine was dependent on each other. In the Δ*yhiF* strain, *ihfA* was no longer downregulated in response to D-serine and similarly in the p*ihfA* strain, *yhiF* levels were comparable with that of the wild type (Figure 3a). These data provide convincing evidence that *yhiF* and *ihfA* co-operate to regulate LEE expression following addition of D-serine. A simple model summarizing these data is presented in Figure 3c.

### Carriage of dsdCXA and the LEE is rare

**Figure 4:**
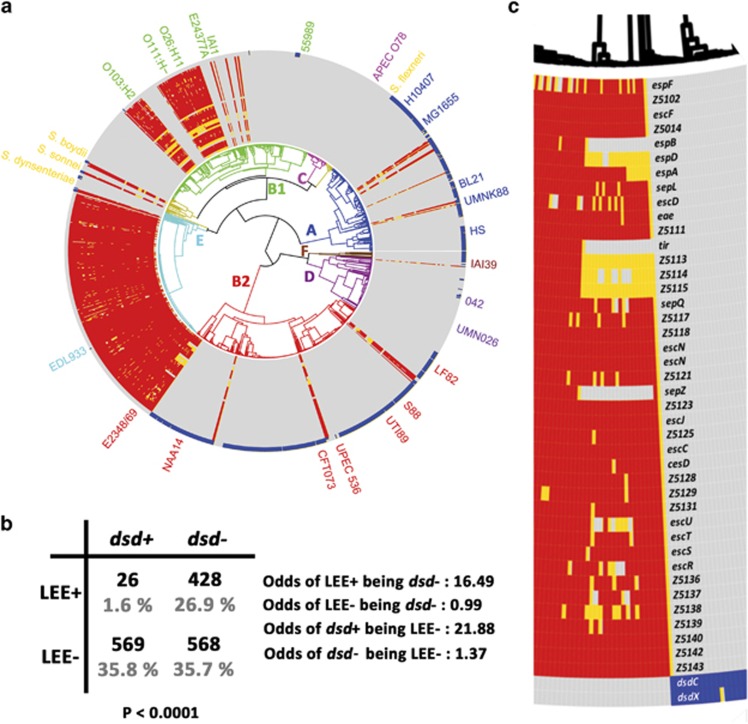
Carriage of both the LEE and *dsdCXA* is rare amongst *E. coli* strains. (**a**) Circularized phylogenetic tree of 1591 *E. coli* and *Shigella* isolates overlaid with gene carriage for the LEE island (>80% identity over >80% of the coding sequence highlighted in red; >50% identity over >50% of the coding sequence highlighted in yellow) and *dsdCX* locus (>80% identity over >80% of the coding sequence highlighted in blue; >50% identity over >50% of the coding sequence highlighted in yellow). Phylogenetic sub-grouping is indicated by branch colour coding as follows: Phylogroup A=Blue; Phylogroup B1=Green; Phylogroup B2=Red; Phylogroup C=Magenta; Phylogroup D=Purple; E=Cyan; Phylogroup F=Brown; *Shigella*=Gold. Key strains are labelled according to their location on the tree. (**b**) 2 × 2 contingency matrix for carriage of the LEE and *dsdCXA* loci. The table is based on the 1591 strains investigated for carryiage of the LEE, *dsdCXA,* both loci or neither loci (a ‘+' denotes presence; a ‘−' denotes absence). A strain is assumed *dsd* positive (+) if it carries coding sequence for *dsdC, dsdX* and *dsdA.* A strain is assumed *dsd* negative (−) if it carries the *dsdCX* truncation. The number of strains for each scenario and their percentage distribution amongst the 1591 strains investigated are given in black and grey, respectively. Statistical significance was calculated using a Fisher's exact test (*P*<0.0001). Odds ratio (OR) of LEE versus *dsdCXA* carriage are indicated also. (**c**) Expansion of LEE+ EPEC isolates from phylogroup B2 exemplifies the clear distinction between LEE and *dsdCXA* carriage.

Our data showed that D-serine had potent effects on the expression and function of the T3SS. This sensitivity to D-serine suggested that strains carrying the LEE would be limited to colonization of environments with low concentrations of this amino acid, hence there would be no requirement to metabolize D-serine and low carriage of the full *dsdCXA* locus. We assessed the frequency of carriage of the LEE and the *dsdCXA* locus in 1591 strains of sequenced *E. coli* isolates available in the NCBI database (as available on 3/6/2014) (Figure 4a). Among strains carrying either the LEE or the *dsdCXA* locus, there was a strong correspondence to absence of the reciprocal locus (odds ratios of 16.49 and 21.88, respectively). In contrast, among LEE negative strains, there was no correspondence with presence or absence of the *dsd* locus (odds ratio 0.99) with a corollary in strains lacking *dsdCX* also being LEE negative (odds ratio 1.37) (Figure 4b). These data demonstrate that for LEE negative strains, there was an equal probability of the *dsd* locus being carried or absent (Figure 4b). In contrast, for strains carrying either the LEE or the *dsdCXA* locus, there was a significant correspondence to absence of the reciprocal locus (odds ratios of 16.49 and 21.88, respectively; *P*<0.0001). These data demonstrate a clear distinction in the carriage of these two loci, which is consistent with them being functionally incompatible.

Analysis of *dsdA* shows that it is highly conserved across the *E. coli* lineage; indeed, over 98% of strains (1561 of 1591) investigated in this study carried *dsdA.* Mapping the nucleotide diversity of *dsdA* results in a phylogenetic tree very similar to one constructed using sequences from the core genome (Supplementary Figure 5). This suggests that *dsdCXA* are ancestral and that the ability to metabolize D-serine was a capacity of the progenitor *E. coli*. Moreover, it is also important to address whether loss of *dsdCX* preceded acquisition of the LEE. Our analysis shows that 1.6% of strains analysed carried both *dsdCXA* and the LEE indicating that the LEE can be acquired in a *dsdCXA* background but that subsequent selective pressures typically result in loss of *dsdCX* (Figure 4b).

To further test our hypothesis, we looked more closely within phylogroup B2 (Figure 4c), represented by 427 strains in our analysis. This phylogroup contains primarily extraintestinal pathogenic *E. coli* strains including the well-characterized UPEC strains 536, UTI89 and CFT073 as well as strain S88, a neonatal meningitis associated isolate. Notably, B2 also contains a number of intestinal pathogenic *E. coli* strains that provide an informative comparator. The vast majority of B2 strains (344/427=81%) carried *dsdCXA*. Strikingly, the enteropathogenic *E. coli* (EPEC) strains within this phylogroup, representing the majority of the intestinal pathogenic *E. coli* strains, all lack *dsdCX,* but carry the LEE (Figure 4c). This is interesting as these EPEC strains are more closely related to the extraintestinal pathogenic *E. coli* isolates phylogenetically, but have undergone two clear genetic changes that correspond with adaptation to a strictly gastrointestinal tract niche. This highlights the powerful selective pressure to lose *dsdCX* when the LEE is acquired.

## Discussion

Understanding the basis by which a pathogen targets a particular niche is critically important when designing potential intervention strategies such as vaccines, probiotics and anti-virulence agents. Furthermore, even when we have a good understanding of the molecular basis to pathotype tropism, predicting how evolutionary pressures will shape emergent strains is challenging. For example, the 2011 outbreak of *E. coli* O104:H4 was caused by an outbreak strain carrying virulence genes for Shiga toxin production and aggregative adherence to intestinal epithelial cells, a previously rare combination (Bielaszewska *et al.*, 2011; Karch *et al.*, 2012). On this basis, we have considered why *E. coli* O157:H7 is generally limited to the gastrointestinal tract, and why it is rarely associated with the colonization of other niches. Comparison with UPEC isolates is particularly informative as these strains compete well both in the gastro-intestinal tract and are capable of extraintestinal disease (Chen *et al.*, 2013).

Previous work has demonstrated that the *dsdCXA* locus is carried extensively by UPEC isolates. Indeed, the ability to metabolize D-serine is entirely compatible with strains that infect the bladder as urine contains ∼28 to 1 mM of D-serine (Anfora *et al.*, 2007). Thus, the catabolism of D-serine represents a positive *E. coli* fitness trait during urinary tract infection. Our data have shown that the concentration of D-serine in the gastrointestinal tract of mice to be far lower than this, at ∼1 μM. On the basis of this, our working model is that LEE-positive strains are largely restricted to the gastro-intestinal tract as this environment has insufficient D-serine concentrations required to block expression of the LEE. In contrast, distal sites such as the bladder contain concentrations of D-serine that would block LEE mediated adhesion.

D-isomers of AAs provide not only potential carbon sources but have also been shown to be important regulatory signaling molecules (Cava, Lam, *et al.*, 2011). On the basis of this, we postulated that D-AAs might be important in the regulation of virulence genes in *E. coli* O157:H7. Testing a variety of D-AAs showed that D-serine selectively affected LEE gene expression. Whereas D-serine can be bacteriostatic in minimal medium at ∼0.475 mM (Cosloy and McFall, 1973; Nørregaard-madsen and Fall, 1995), addition of up to 3 mM D-serine did not affect the growth rate of *E. coli* O157:H7 grown in minimal essential media (MEM-HEPES) (data not shown). This media is typically used for culture of O157:H7 as it normally induces expression of the T3SS (Tree *et al.*, 2011).

Remarkably, addition of D-serine reduced T3SS expression by modulating the expression of the majority of the LEE genes. The resulting phenotype was clear: strong reduction in the secretion of T3SS effector proteins and reduced attachment to host cells. Although expression of the LEE was reduced, D-serine also caused a selective upshift in gene expression. Strikingly, exposure to D-serine resulted in an upregulation of 21 genes of the SOS-regulon. This ‘stress' response was confirmed by immunoblotting for RecA and qRT-PCR.

The induction of the SOS response was interesting as it is normally associated with a global response to DNA damage (Kenyon and Walker, 1980; Michel, 2005). However, induction of an SOS response can be achieved by alternative mechanisms, for example, by addition of β-lactams that inhibit the *ftsI* gene product, penicillin binding protein 3 (Maiques *et al.*, 2006; Miller *et al.*, 2013). Previous work has shown that *E. coli* can covalently link certain D-AAs to its peptidoglycan and that in the case of *V. cholerae*, cause a rod-to-sphere transition (Lam *et al.*, 2009). The exact mechanism by which D-serine induces the SOS response will be pursued as a separate study, but it is exciting to speculate that this may be linked to changes in peptidoglycan cross-linking as we also observed more spherical cells upon addition of D-serine to the growth media (unpublished data).

As D-serine supplemented in the growth environment resulted in induction of the SOS response and repression of the LEE, we tested whether these responses were co-dependent. Transformation of O157:H7 with a plasmid (p*dsdA*) expressing D-serine deaminase from UPEC, allowed the strain to metabolize D-serine and restored RecA expression to the same level as seen in the absence of D-serine (Figure 2b). In contrast, analysis of the secreted protein profile and immunoblotting for EspD showed that even when O157:H7 can metabolize D-serine, the T3SS is inhibited and expression of effector proteins is reduced (Figure 2b). These data show that D-serine causes repression of the T3SS even in the absence of the SOS response. Indeed, inspection of our transcriptomic analysis revealed that two known regulators of the LEE were affected by addition of D serine: YhiF and IhfA (Friedberg *et al.*, 1999; Tatsuno *et al.*, 2003; Tree *et al.*, 2011). IhfA is the alpha-subunit of the IHF and is a member of the nucleoid-associated proteins, which are involved in a number of cellular processes including transcriptional regulation (Goosen and van de Putte, 1995). The involvement of IHF in multiple regulons has been explored recently in *E. coli* K-12, revealing over 30% of all predicted operons containing IHF-binding regions and ∼10% of these showing differential expression in an *ihfAB* double mutant (Prieto *et al.*, 2012). Specifically, IHF has also been shown to play a critical and direct role in positive regulation of the LEE1 promoter, *via* the Ler master regulator which subsequently activates operons LEE2 to LEE5 (Friedberg *et al.*, 1999). The second regulator identified, YhiF, is a member of the GAD acid stress response regulators which have been identified as negative regulators of the LEE2 and LEE5 promoters (Tatsuno *et al.*, 2003). In line with this knowledge, we identified that both regulators are differentially expressed to promote repression of the LEE. Interestingly, IhfA and YhiF were found to be co-operative in response to D-serine suggesting an interplay that leads to repression of the LEE *via* these two regulators (Figure 3a). Constitutive expression of *ihfA* removed the repression of the LEE and restored *yhiF* levels to that of the wild type suggesting that YhiF may repress *ihfA* at the transcriptional level. Similarly, deletion of *yhiF* restored wild-type *ihfA* expression levels and removed LEE repression activated by D-serine. We propose that IHF and YhiF form a regulatory feedback loop in which repression of *ihfA* downregulates LEE1 expression and upregulation of *yhiF* represses the LEE further at the LEE2 and LEE5 operons (Figure 3c). Regulators of the GAD acid stress response have previously been shown to be overridden by alternate regulatory systems to modulate the LEE (Tree *et al.*, 2011; Branchu *et al.*, 2014), so it is conceivable to imagine a D-serine-specific response system that is mediated through key regulators of the LEE. However, despite this evidence that IhfA and YhiF regulate the LEE in response to D-serine, whether this amino acid acts directly on these regulators remains to be tested.

Exposure of *E. coli* O157:H7 to D-serine caused a distinct pattern of gene expression. We postulated that the importance of this might affect carriage of the *dsdCXA* locus for two rather distinct reasons. Firstly, a functional *dsdA* gene did not overcome the repression of the LEE—such that the T3SS is not functional when D-serine is present in the environment. This suggested that carriage of *dsdCXA* would offer little advantage for pathogens with the LEE. Secondly, a functional *dsdA* gene prevented the SOS response thereby fundamentally changing the gene expression profile of the pathogen. To test this, we assessed the frequency of carriage of LEE and *dsdCXA* genes in 1591 strains of sequenced *E. coli* isolates and showed a strong distinction in the carriage of the LEE and *dsdCXA*, which is consistent with them being functionally incompatible. In place of *dsdCX,* most strains contain the locus for sucrose utilization (*cscBKAR*), an observation that has been made for some *E. coli* K12 strains in the 1970s (Alaeddinoglu and Charles, 1979). Insertion of the sucrose operon is facilitated by the presence of the *argW* gene, which codes for an arginine-specific tRNA and acts as a hotspot for recombination (Jahreis *et al.*, 2002). Clearly, the ability to metabolize sucrose provides a tangible selective advantage to strains carrying this operon but, in addition, the concurrent loss of *dsdCX* affects the gene expression response of *E. coli* O157:H7 to D-serine. Some rare isolates (26 from 1591 genomes analysed) appear to carry both the LEE and *dsdCXA*. These strains were all isolated from stool samples suggesting they would be classified as intestinal pathogenic *E. coli*. Our interpretation is that these strains have acquired the LEE without opportunity for subsequent selective pressures to result in replacement of *dsdCXA*.

As D-serine affects expression of the LEE, it seems likely that highlights that this amino acid provides an important signal as to where and when to attach to host tissue. Sites commonly infected by extraintestinal pathogenic *E. coli* strains such as the bladder and meninges both contain high levels of D-serine (Wolosker *et al.*, 2008) and we have shown that the LEE is rarely carried in these pathotypes as it does not function when in physiologically relevant concentrations of this amino acid. It should be noted that some rare, but serious, cases of urinary tract infection have resulted from Shiga toxin positive strains, although genetic information on their gene context is very sparse (Tarr *et al.*, 1996). Interestingly, the archetype *E. coli* K1 neonatal meningitis strain, strain RS218, has two copies of the *dsdCXA* genes (Moritz and Welch, 2006), facilitating infections in this tissue. Fascinatingly, one NMEC strain, *E. coli* O7:K1 CE10, has been reported to carry a functional T3SS that is involved in the invasion and intracellular survival in human brain microvascular endothelial cells (Yao *et al.*, 2009). However, this strain does not carry the LEE but instead appears to have a functional ‘second type' or ETT2 secretion system that delivers effector proteins into host cells. On the basis of our data, we would expect that the CE10 ETT2 system is functional in D-serine and regulated very differently compared with the LEE. In this way, the NMEC strain has adapted to tolerate and metabolize D-serine whilst still retaining a functional delivery system for effector proteins. Overall, our study provides novel insights into how a single amino acid affects gene regulation in *E. coli* O157:H7 and that this can have stark implications for the niche specificity of this pathotype.

## Notes

The authors have confirmed that they have no financial, personal or professional interests that could be construed to have influenced the paper.
