# Supplementary figures and images for: The host metabolite D-serine contributes to bacterial niche specificity through gene selection
Source: ISME J. 2014 Dec 19;9(4):1039–51. doi: 10.1038/ismej.2014.242 (PMC4366372; doi:10.1038/ismej.2014.242)

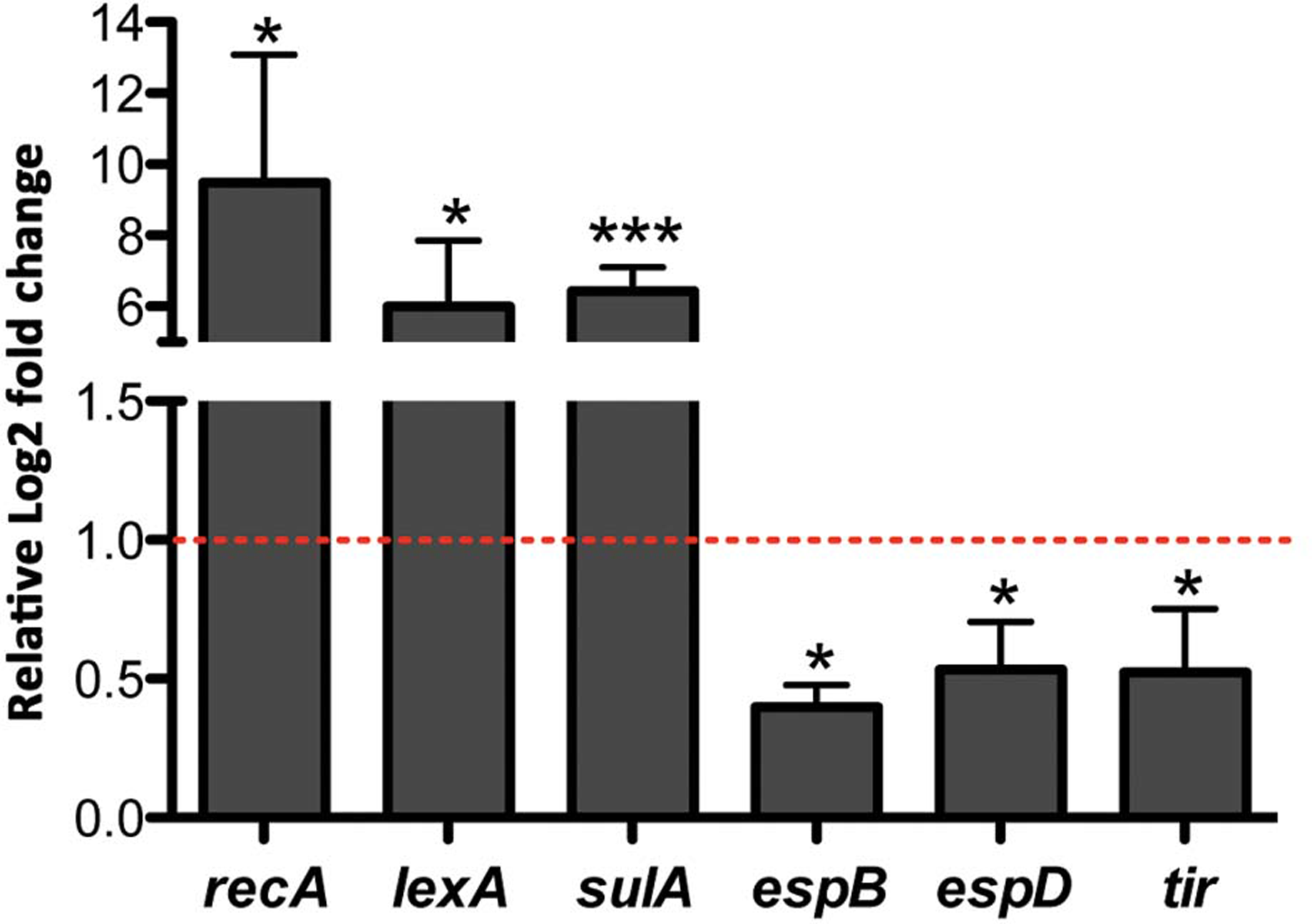

Supplement: Supplementary Figure 1 [file ismej2014242x2.tif]

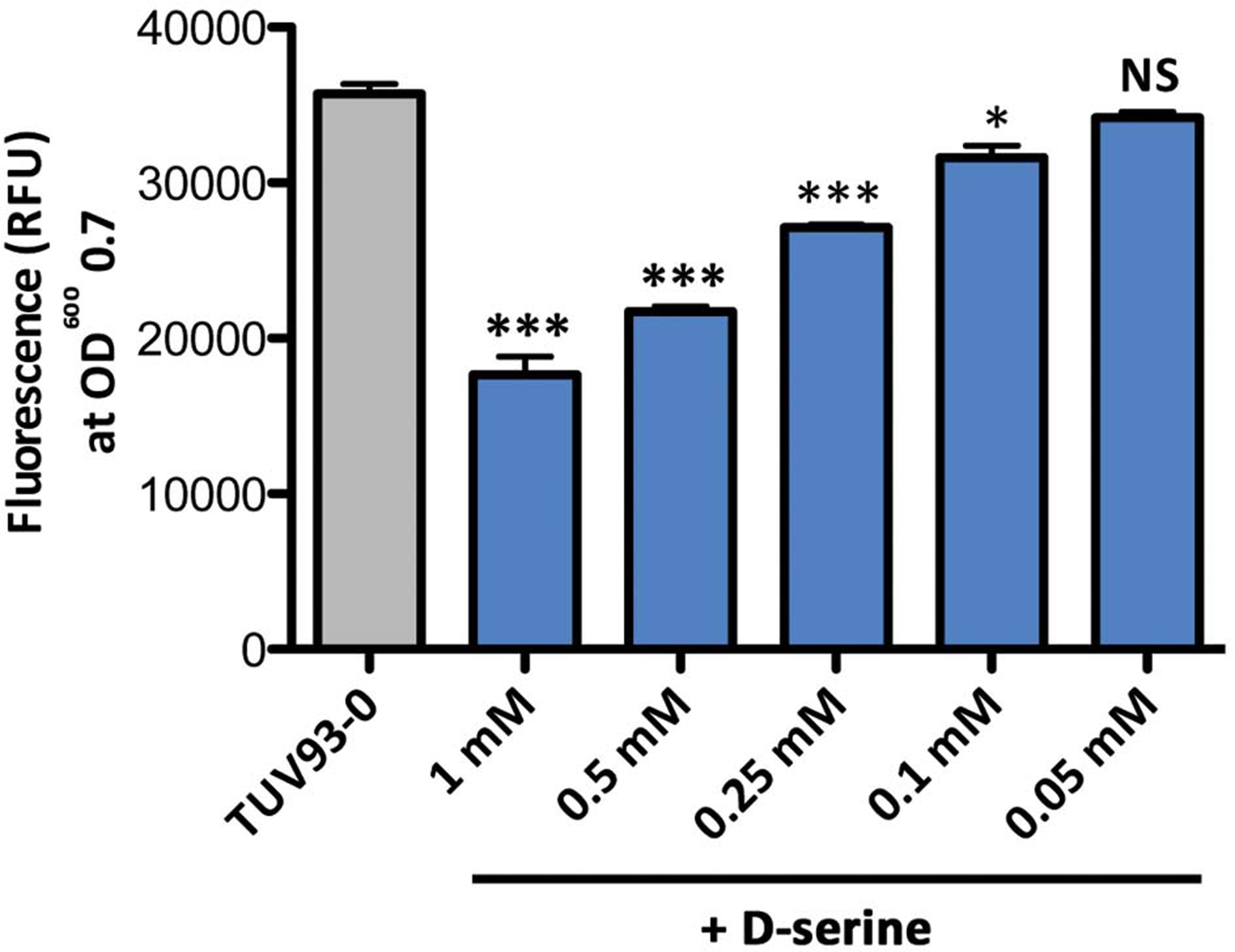

Supplement: Supplementary Figure 2 [file ismej2014242x3.tif]

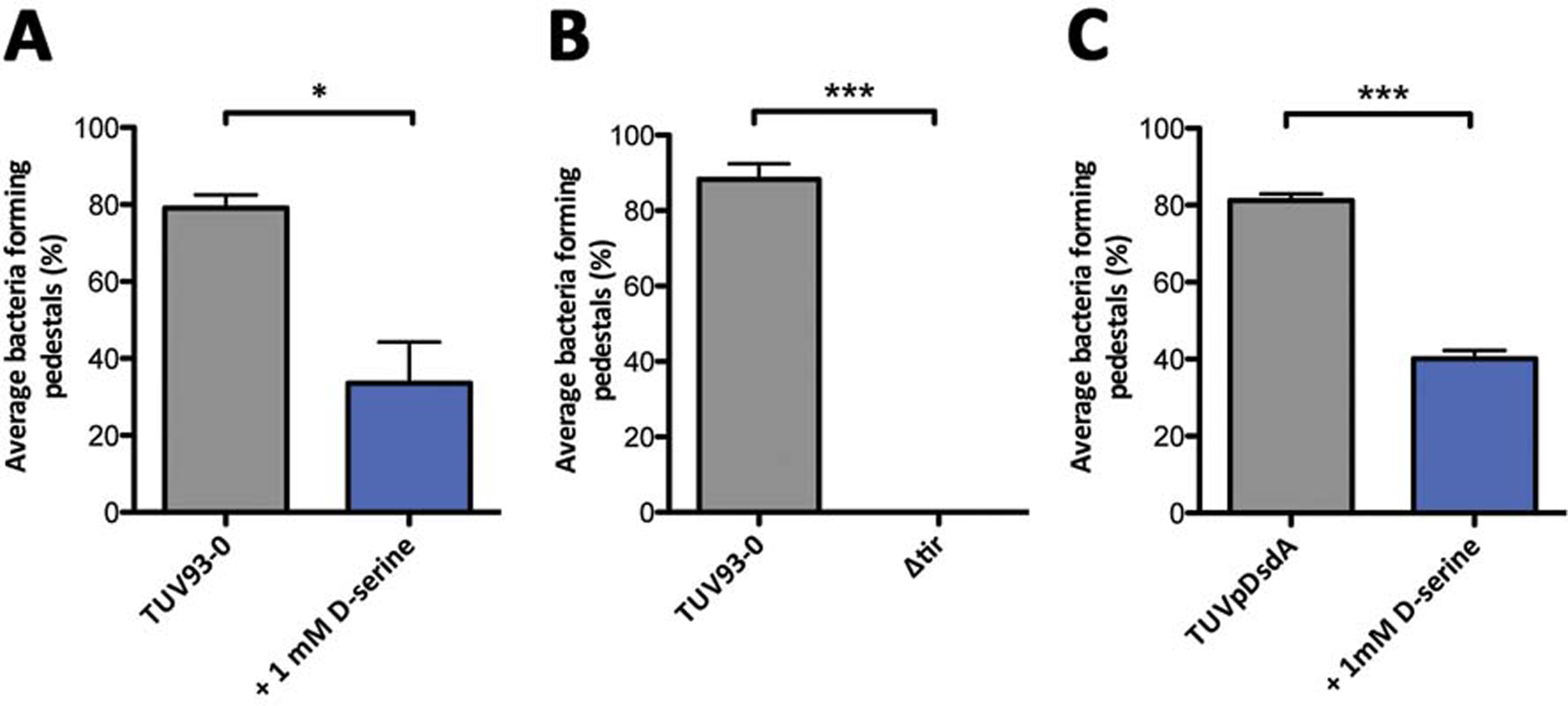

Supplement: Supplementary Figure 3 [file ismej2014242x4.tif]

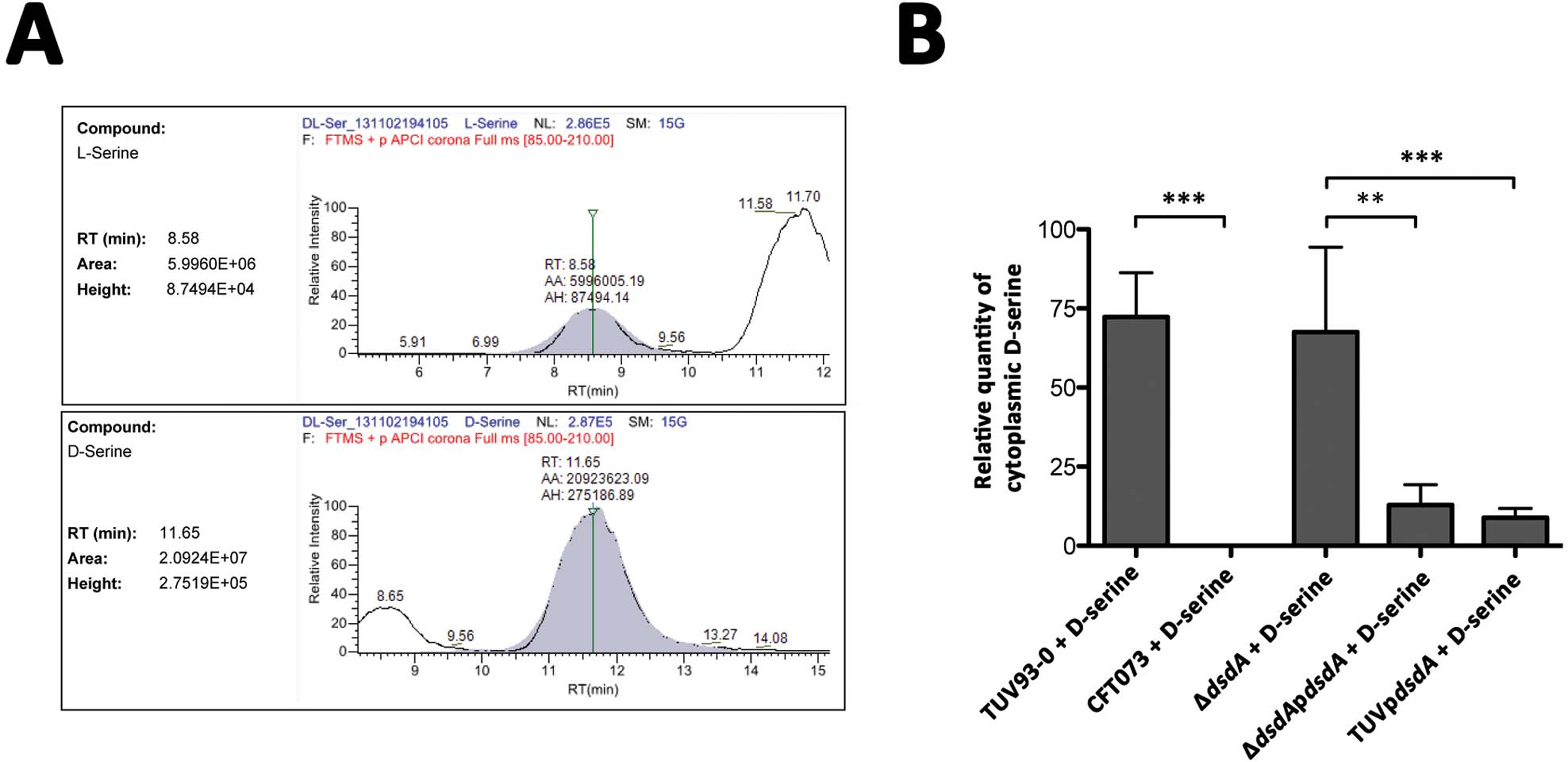

Supplement: Supplementary Figure 4 [file ismej2014242x5.tif]

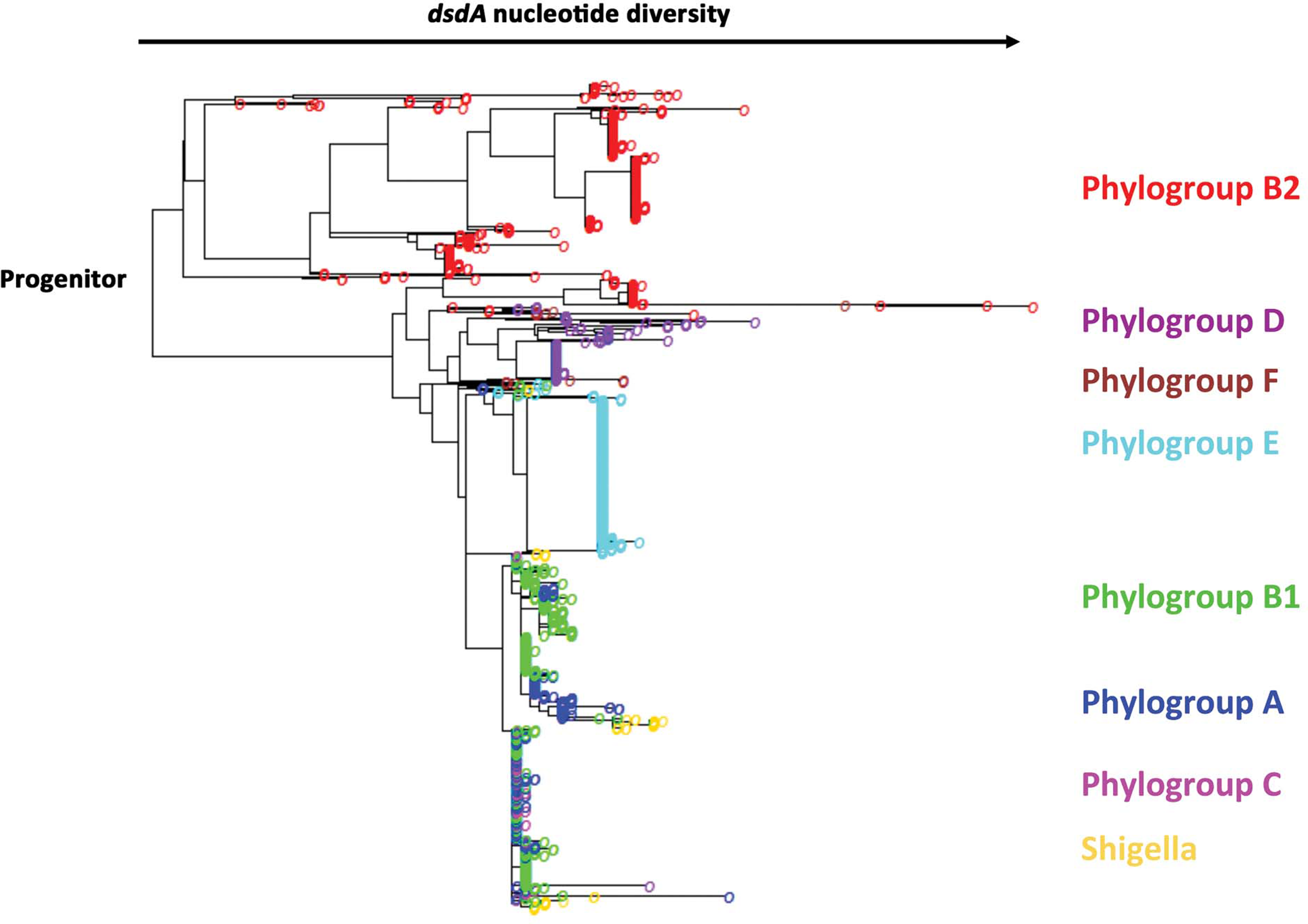

Supplement: Supplementary Figure 5 [file ismej2014242x6.tif]
