# Supplementary material for: The host metabolite D-serine contributes to bacterial niche specificity through gene selection
Source: ISME J. 2014 Dec 19;9(4):1039–51. doi: 10.1038/ismej.2014.242 (PMC4366372; doi:10.1038/ismej.2014.242)
Supplement: Supplementary Figure Legends [file ismej2014242x7.doc]

**Supplementary Title and Figure Legends**

**Supp. Fig. 1. qRT-PCR validation of RNA-seq data**

qRT-PCR validation of LEE repression (*espB, espD* and *tir* in response to D-serine identified by RNA-seq. The red dashed line indicates relative baseline expression, with genes expressed above this being up-regulated and genes expressed below this being down-regulated. * and *** denote *p* ≤ 0.05 and *p* ≤ 0.001 respectively calculated from 3 biological replicates.

**Supp. Fig. 2. Concentration dependent effects of D-serine on LEE1 expression**

Screening the effects of 1 mM, 0.5 mM, 0.25 mM, 0.1 mM and 0.05 mM D-serine on LEE1 expression using a GFP tagged LEE1 promoter fusion reporter (PLEE1:GFP). *** and * denotes P ≤ 0.001 and P ≤ 0.05 respectively calculated from 3 biological replicates. Relative Fluorescence Units (RFU) were derived from a standard curve of Optical Density at 600 nm (OD^600^) measured over time. Bacteria were cultured in MEM-HEPES to promote expression of the T3SS.

**Supp. Fig. 3. Analysis from EHEC and UPEC cell-adhesion assays.**

Histograms displaying the average bacteria forming actin pedestals (A/E lesions) during cell-adhesion assays, with and without exposure to D-serine in a wild type TUV93-0 background (**A**), a TUV93-0 Δ*tir* background (**B**) and a TUVp*dsdA* background (**C**). * and *** denote *p* ≤ 0.05 and *p* ≤ 0.001 respectively calculated from 3 biological replicates.

**Supp. Fig. 4. Intracellular accumulation of D-serine in strains lacking *dsdA*.**

(**A**) Chromatographic peaks of standard L-serine and D-serine showing differential retention times as measured by LC-MS. This standard allows for relative quantification of D-serine from whole cell metabolite pools. (**B**) Relative quantity (%) of D-serine obtained from whole cell metabolite pools of TUV93-0, UPEC CFT073, UPEC Δ*dsdA,* UPEC Δ*dsdA*p*dsdA* and TUVp*dsdA* cultured in MEM-HEPES supplemented with 1 mM D-serine. *, ** and *** denote *p* ≤ 0.05, *p* ≤ 0.01 and *p* ≤ 0.001 respectively calculated from 3 biological replicates.

**Supp. Fig. 5. Ancestry of *dsdA.***

Phlyogenetic tree generated based on maximum likelihood of *dsdA* sequence diversity. Phylogenetic sub-grouping is indicated as follows: Phylogroup A = Blue; Phylogroup B1 = Green; Phylogroup B2 = Red; Phylogroup C = Magenta; Phylogroup D = Purple; Phylogroup E = Cyan; Phylogroup F = Brown; *Shigella* = Gold.
